# Potentiation of Catalase-Mediated Plant Thermotolerance by N-Terminal Attachment of Solubilizing/Thermostabilizing Fusion Partners

**DOI:** 10.3390/ijms252212181

**Published:** 2024-11-13

**Authors:** Guoqing Xie, Yanrong Huang, Di Hu, Yinyu Xia, Ming Gong, Zhurong Zou

**Affiliations:** Engineering Research Center of Sustainable Development and Utilization of Biomass Energy, Ministry of Education, School of Life Sciences, Yunnan Normal University, Kunming 650500, China; xgq7779@163.com (G.X.); hyr2223120023@163.com (Y.H.); hudihey@foxmail.com (D.H.); xyy2969924153@163.com (Y.X.); gongming63@163.com (M.G.)

**Keywords:** catalase, protein solubility, protein thermostability, protein fusion expression, thermotolerance, hyperacidic mini-peptide, rubredoxin

## Abstract

Catalase (CAT) plays a crucial role in plant responses to environmental stresses and maintaining redox homeostasis. However, its putative heat lability might compromise its activity and function, thus restricting plant thermotolerance. Herein, we verified Arabidopsis CAT3 was of poor thermostability that was then engineered by fusion expression in *Escherichia coli*. We found that our selected fusion partners, three hyperacidic mini-peptides and the short rubredoxin from hyperthermophile *Pyrococcus furiosus*, were commonly effectual to enhance the solubility and thermostability of CAT3 and enlarge its improvement on heat tolerance in *E. coli* and yeast. Most importantly, this finding was also achievable in plants. Fusion expression could magnify CAT3-mediated thermotolerance in tobacco. Under heat stress, transgenic lines expressing CAT3 fusions generally outperformed native CAT3 which in turn surpassed wild-type tobacco, in terms of seed germination, seedling survival, plant recovery growth, protection of chlorophyll and membrane lipids, elimination of H_2_O_2_, as well as mitigation of cell damage in leaves and roots. Moreover, we revealed that the introduced CAT3 or its fusions seemed solely responsible for the enhanced thermotolerance in tobacco. Prospectively, this fusion expression strategy would be applicable to other crucial plant proteins of intrinsic heat instability and thus provide an alternative biotechnological route for ameliorating plant heat tolerance.

## 1. Introduction

Catalase (CAT) is a core antioxidative enzyme containing the heme cofactor in plants, catalyzing the decomposition of H_2_O_2_ into water and oxygen, thereby controlling the abundance of this important ROS signaling molecule and maintaining cellular redox balance during plant growth, development, and adaptations to environmental stresses [1]. In plant cells, CAT protein is highly conserved in a tetramer structure with each monomer of 50–70 kDa [2], and abundantly localized in peroxisomes known as the hub of intracellular H_2_O_2_ production, metabolism, and signal transduction [3]. The entry of CAT into peroxisomes may involve a non-classic import pathway using peroxisome targeting signal 1 (PTS1) [4].

Plant CAT is usually encoded by small gene families. In Arabidopsis, the CAT family has three members with nearly identical sequences, i.e., cytosolic CAT1 and peroxisomal CAT2 and CAT3. The spatiotemporal patterns and regulatory mechanisms of their gene expression are different, reflecting their diverse roles in plant physiological responses [5]. In peroxisomes, glycolate oxidase within the photorespiration pathway is the main trigger to produce H_2_O_2_ in the leaves of C3 plants (e.g., rice) that can be directly quenched by the structure-associated CAT enzymes, indicating an essential role of CAT in photorespiration [6]. CAT is also engaged in plant development, especially during the processes with obvious increases in intracellular ROS such as organ aging [7]. CAT is strictly associated with autophagy elicited by developmental signals and environmental stresses. Peroxisome aggregates and enhanced pexophagy can be observed in CAT-deficient mutant plants [8]. In addition, changes in CAT activity or expression are also related to plant immunity underlying resistance to pathogen infections [9]. Additionally, CAT is tightly connected to plant responses and resistance to abiotic stresses (e.g., drought, high salinity, high light, and heat) that usually upregulate its expression [10,11]. Meanwhile, during these involvements, CAT activity is responsively modulated, mainly by posttranslational modifications (PTMs) such as phosphorylation, glycosylation, carbonylation, nitrosylation, persulfidation, acetylation, succinylation, etc. [1,3,12,13,14].

Among those CAT-involving abiotic stresses, high temperature severely impacts on crop productivity by causing cell membrane damage, ROS accumulation, photosynthesis decay, metabolism disorders, and plant hormone imbalances [15]. Abundant evidence has revealed the indispensable role of CAT in the development of antioxidation-directed thermotolerance in plants [16]. For instance, the immune activator ENHANCED DISEASE SUSCEPTIBILITY 1 in rice (OsEDS1) stimulates CAT activity through its association with catalase C (OsCATC), thereby promoting H_2_O_2_ scavenging and activating rice heat tolerance. Its loss-of-function mutant is more sensitive to high temperatures [17]. Similarly, the double-stranded RNA-binding protein SRL10 directly interacts with catalase B (OsCATB), mutually increasing their stability to enhance H_2_O_2_ elimination and consequently contributing to thermotolerance and stable yield under heat stress in rice. The srl10 mutant has an elevated sensitivity to high temperature [18]. In tomato, ectopic expression of Arabidopsis glutaredoxin AtGRXS17 enhances thermotolerance by increasing CAT enzyme activity and reducing H_2_O_2_ accumulation, concomitantly with upregulated expression of the heat shock transcription factor (HSF) and heat shock protein (HSP) [19]. In Chinese cabbage, transient overexpression of the WRKY transcription factor BcWRKY22 directly activates the expression of catalase 2 (BcCAT2) and enhances CAT enzyme activity to diminish H_2_O_2_ under heat stress, and its heterologous introduction also promotes thermotolerance in Arabidopsis [20]. In addition, 2C-type protein phosphatase (PP2C) negatively regulates thermotolerance in cassava by directly dephosphorylating CAT1, which then has lower H_2_O_2_-scavenging activity [21]. Overexpression of annexin OsANN1 enhances heat stress tolerance in rice by promoting CAT and superoxide dismutase (SOD) activities that regulate H_2_O_2_ content and redox homeostasis, probably through phosphorylation as OsANN1 interacts with the kinase OsCDPK24 [22].

Nevertheless, CAT is inferred to have intrinsic instability in a few previous studies. The folding, oligomerization, stabilization, and activation of CAT enzymes may require the assistance of some interacting proteins such as LESION SIMULATING DISEASE1 (LSD1) [23], OsEDS1 [17], SRL10 [18], TaWD40-4B.1 [24], chaperones (e.g., NO CATALASE ACTIVITY1 (NCA1) [25], Hsp17.6CII [26]), or other heat shock factors [19]. Hsp17.6CII can also independently inhibit the aggregation of CAT2 protein in vitro [26]. In addition, the main catalases (CAT2, CAT3) in Arabidopsis largely accumulate as aggregates under heat stress in the proteostasis-impaired *chip* or *nbr1* single mutant and, to a greater extent, in the *chip nbr1* double mutant, with a parallel loss of activities. These mutants also exhibit compromised heat tolerance [27]. Therefore, the heat lability of CAT might restrict plant thermotolerance, and how to improve the thermostability of plant CAT proteins is certainly of interest to enhance plant resistance to high temperatures.

Nowadays, numerous mutagenesis-based approaches and their updates with computer design or AI assistance have been prevalently used for protein stability engineering [28,29], but usually encounter a complex selection process and must overcome the stability–function trade-off in proteins [30]. Therefore, the protein fusion strategy with obvious simplicity, considerable efficacy, and minimal negative effects also deserves attention [28,31]. Fusing with a partner with high stability can improve the thermostability and activity of target proteins under high-temperature conditions [32,33,34], and this effect can also be achieved by fusion-mediated enhancement of protein solubility [35]. Both of these scenarios also occurred in our previous studies. We have validated that the short rubredoxin from hyperthermophile *P. furiosus* (PfRub) and some hyperacidic mini-peptides (HAMPs) can be used as fusion partners (or tags) to enhance the solubility, thermostability, and activity of several target proteins as well as the heat resistance of their recombinant microbial strains [36,37,38,39]. Rubredoxin is a small Fe-S protein (around 53 amino acids) and usually acts as an electron donor in many enzymatic reactions [40]. PfRub has been proven to be of extraordinary thermostability (above 100 °C) with a melting temperature close to 200 °C, which depends on its unique protein structure [41]. Its hyperthermostability might be in cis transferable to its fused thermosensitive client proteins, likely stemming from enthalpic and entropic mechanisms [32,36]. PfRub has also been used as a red-colored fusion moiety to visually monitor the soluble expression and the purification process of target proteins due to its Fe^3+^-bound spectral character [42]. HAMPs are highly rich in acidic amino acid residues and can endow fused proteins with a large amount of negative charge. The repulsive force of the same charge is accordingly strengthened and antagonizes protein molecules to condensate, thus increasing solubility and thermostability [37,38,39,43]. HAMPs perhaps also possess an intramolecular chaperoning activity to benefit the solubility of client proteins [44].

Herein, we again utilized these thermostabilizing/solubilizing tags through fusion expression to enhance the thermostability and activity of plant CAT proteins under high temperatures, thereby improving plant tolerance to heat stress. Our results verified the fragile thermostability of Arabidopsis CAT3. N-terminal fusion expression of CAT3 with selected tags not only enhanced its thermostability with relatively less activity loss under heat but also magnified its promotion of heat tolerance in recombinant microbial (*E. coli*, yeast) strains and particularly in transgenic tobaccos. This protein-engineering strategy might have application potential in developing climate-resilient crops to fulfill sustainable agriculture needs.

## 2. Results

### 2.1. Fusion Expression Enhances the Solubility and Thermostability of Arabidopsis CAT3 and Protects It from Heat Inactivation

Arabidopsis CAT3 was previously inferred to be thermolabile [26,27] but this was never substantially confirmed. Herein, we cloned its CDS region by reverse transcription PCR (RT-PCR) and constructed its prokaryotic expression vector pET(CAT3) with the backbone of pET32a(+). Subsequently, we introduced a set of solubilizing/thermostabilizing fusion partners (ra3t (r), tua2 (t), p60c (p), and rub (u) (i.e., PfRub)) and created a series of N-terminal fusion expression vectors, pET(r/t/p/uCAT3). The properties of fusion partners, CAT3, and its fusion proteins are listed in Table 1. Fusion was designated at the N-terminal to ensure little disturbance on the import of CAT3 into peroxisomes of transgenic plants as described below.

These prokaryotic vectors were transformed in *E. coli* strain BL21(DE3) for recombinant expression of CAT3 and its various fusion proteins (r/t/p/uCAT3) by IPTG induction. As shown by SDS-PAGE analysis, all forms of CAT3 proteins were evidently expressed with correct sizes. The non-fused CAT3 protein was expectedly of poor solubility (approximately 10%) even under expression at low temperature (20 °C), with the majority in the insoluble inclusion bodies. In contrast, various CAT3 fusion proteins (rCAT3, tCAT3, pCAT3, uCAT3) were expressed with a significantly higher solubility level (around 50%) (Supplementary Figure S1). This solubility enhancement would be certainly ascribed to the fusion partners (ra3t, tua2, p60c, and rub) attached to CAT3.

**Figure 1:**
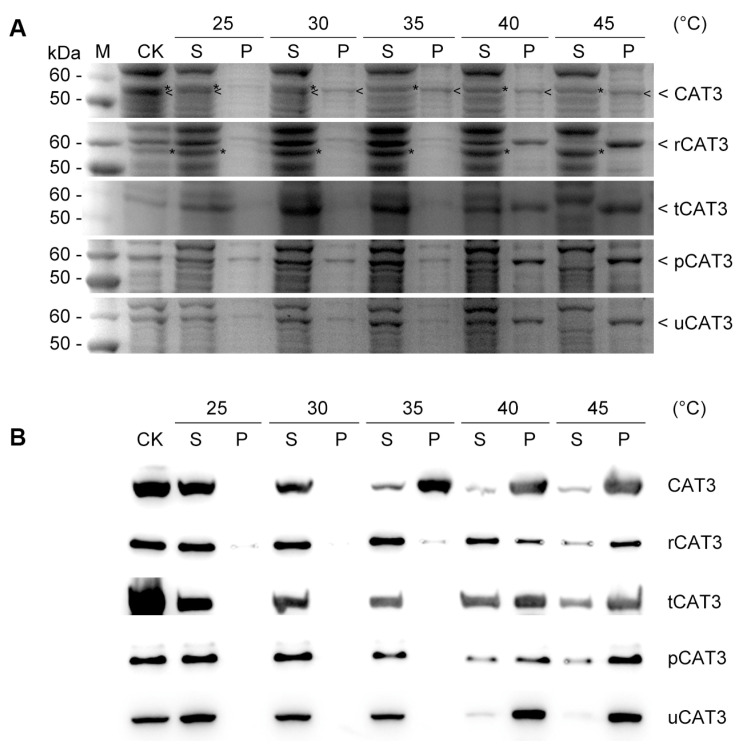
Thermostability of CAT3 and its fusion forms judged by protein solubility changes with increasing temperature, through (**A**) SDS-PAGE analysis and (**B**) immunoblotting detection using a commercial myc-tag antibody on heat-treated supernatant of the bacterial cell lysate expressing CAT3 or its fusion protein (r/t/p/uCAT3). M: protein size marker; CK: non-heat-treated; S, P: the supernatant and pellet of each heat-treated sample fractionated by centrifugation, respectively. ‘<’ indicates the recombinant CAT3 and its fusion proteins. ‘*’ indicates the *E. coli* endogenous protein close to CAT3.

Considering the risk of losing the cofactor heme during purification, we directly subjected the supernatant of expressed bacterial cell lysates to a gradient heat treatment (25–45 °C, 5 °C interval) to appraise CAT3 thermostability. After 30 min of heat incubation, the non-fused CAT3 protein began to precipitate at 30 °C and almost became insoluble by 35 °C. However, all fusion forms of CAT3 maintained solubility till 35 °C, started to show a moderate precipitation at 40 °C, and the majority ultimately became insoluble at 45 °C, indicating that all tested fusion partners (ra3t, tua2, p60c, and rub) significantly improve CAT3 thermostability with a rise in temperature of at least 5 °C (Figure 1A).

We further performed immunoblotting to evaluate the effects of fusion partners on CAT3 thermostability. In our constructs, all forms of CAT3 proteins were added with a myc-tag at the N-terminal (Table 1). After SDS-PAGE of heat-treated samples as aforementioned, the proteins on gels were blotted on membranes for specific detection with a common myc-tag antibody. We found the blot results showing the fluctuation tendency of solubility with increasing temperature were overall consistent with the SDS-PAGE band analysis (Figure 1A). The non-fused CAT3 was truly heat labile and sharply became insoluble at 35 °C, whist the CAT3 fusion forms showed no apparent solubility changes during this heat treatment and maintained a moderate solubility at 40 °C and even higher temperatures (Figure 1B). Collectively, these results demonstrate that the N-terminal attachment of effectual fusion partners can significantly improve the solubility and thermostability of CAT3 heterologously expressed in *E. coli*.

**Figure 2:**
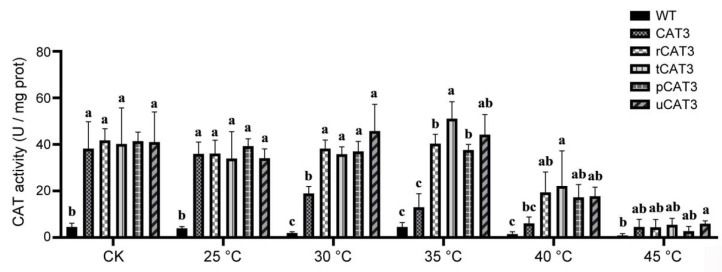
The enzymatic activity changes with increasing temperature of CAT3 and its fusion proteins (r/t/p/uCAT3) recombinantly expressed in *E. coli*. CK: non-heat-treated. Variance significance (a, b, c) was defined according to the Duncan test method.

Meanwhile, we also monitored the enzymatic activities of all recombinant CAT3 proteins during the gradient heat treatment, using the supernatant of induced *E. coli* cell lysates to avoid loss of the essential cofactor heme. After heat incubation for 30 min at each temperature, the entire samples were immediately assayed for CAT activities without fractionation. The baseline activity of endogenous catalases in wild-type (WT) *E. coli* was used as a reference. We found that the heterologous overexpression of all CAT3 forms exhibited about a ten-fold increase in CAT activity than WT. During heat treatment, the enzymatic activity of non-fused CAT3 dropped significantly at 30 °C, dramatically (nearly a quarter remained) at 35 °C, and to a severe degree of less than 10% at 40 °C, as compared to untreated control (CK). In contrast, all CAT3 fusions consistently maintained activity or had even higher activity below 35 °C and began to show a moderate activity decay (to nearly half) at 40 °C. Finally, at 45 °C, all forms of CAT3 were inactivated, displaying a residual activity similar to the background value of untreated WT (Figure 2). This enzymatic activity change with heat was overall in accordance with the thermostability dynamics described above for all CAT3 forms, confirming that only soluble CAT proteins possess catalytic activity. This result also indicates that our tested fusion partners can remarkably protect its client protein CAT3 from heat inactivation (with the threshold temperature rise of more than 5 °C), besides a concomitant role in enhancing the solubility and thermostability.

### 2.2. Fusion Expression Potentiates CAT3-Mediated Thermotolerance in Its Recombinant E. coli and Yeast Strains

**Figure 3:**
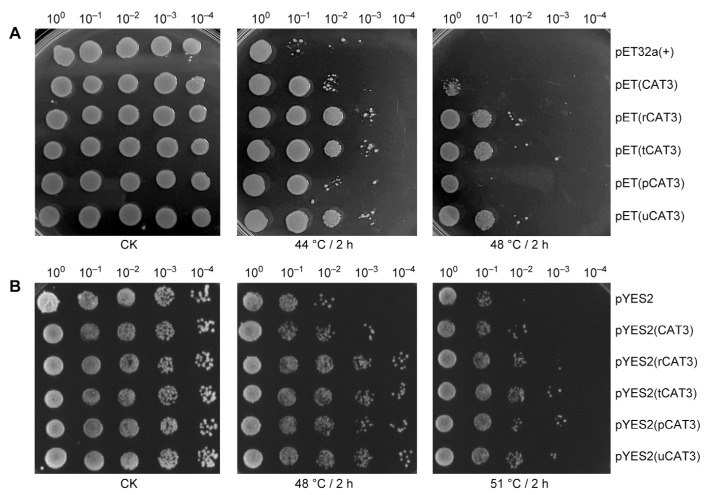
Potentiation of CAT3-mediated thermotolerance in *E. coli* and yeast by capping solubilizing/thermostabilizing fusion partners, as appraised in their recombinant strains by comparing the colony growth status. (**A**) The induced *E. coli* cells under normal conditions (CK) or a period (2 h) of preheating at 44 °C and 48 °C, via the dot-plating test with serial dilutions (1-, 10-, 100-, 1000-, 10,000-fold) on solid LB medium, using the control strain of empty vector pET32a(+). (**B**) The induced *S. cerevisiae* cells under CK conditions or a period (2 h) of preheating at 48 °C and 51 °C, via the dot-plating test with serial dilutions (1-, 10-, 100-, 1000-, 10,000-fold) on solid YPD medium, using the control strain of empty vector pYES2.

CAT is involved in the responses and adaptations of plants to numerous abiotic stresses [10,11]. Herein, we explored the impact of enhanced CAT enzyme activity on the thermotolerance of *E. coli* and yeast *Saccharomyces cerevisiae* overexpressing Arabidopsis CAT3 and its fusion forms by a colony dot-plating test of cell survival after heat stress. As seen in Figure 3A, the colony growth status on solid LB medium of the induced *E. coli* cells was very similar in all strains under normal conditions (CK) but varied upon a treatment of preheating for 2 h at 44 °C and more obviously at 48 °C, i.e., overall, it was slightly better in the *E. coli* strain containing pET(CAT3), moderately better in the fusion protein pCAT3-expressing strain, but much better in those expressing any of the other CAT3 fusion forms (r/t/uCAT3), as compared to the control strain of empty vector pET32a(+). This cell survivorship was overall consistent with the previously evaluated thermostability of CAT3 and its fusion variants recombinantly expressed in *E. coli* (Figure 1).

Afterwards, a serial of CAT3 yeast expression vectors derived from pYES2 were constructed and transferred into *S. cerevisiae* strain INVSC1. As shown in Figure 3B, the colony growth statuses on solid YPD medium of the induced yeast cells were nearly identical in all strains under CK conditions but became notably differential after the recovery from a heat treatment of 2 h at both 48 °C and 51 °C, i.e., generally, a little better in the yeast strain bearing pYES2(CAT3), but predominantly better in those strains expressing any of the CAT3 fusion proteins (r/t/p/uCAT3), as compared to the control strain of empty vector pYES2. This yeast cell survival profile might also be relevant to the thermostability of CAT3 and its fusion forms. Therefore, we examined the solubility changes (for thermostability appraisal) of these recombinant proteins in yeast cells before and after a heat treatment of 2 h at 48 °C by immunoblotting with the antibody of myc-tag (at the N-terminal of all CAT3 proteins). We found all forms of CAT3 heterologously expressed in yeast were almost soluble before heat treatment (CK) but aggregated to different degrees after heat stress, i.e., non-fused CAT3 (nearly half) and r/t/p/uCAT3 fusions (about one-third or less) (Supplementary Figure S2).

Taken together, these results demonstrate that overexpression of CAT3 can confer an improved thermotolerance to both *E. coli* and yeast, and this effect can be largely potentiated (an increase of 3–4 °C) by head-attaching solubilizing/thermostabilizing fusion partners such as ra3t, tua2, p60c, and rub to promote CAT3 thermostability and activity maintenance under heat, making *E. coli* and yeast cells survive at higher temperatures.

### 2.3. Fusion Expression Magnifies CAT3-Mediated Thermotolerance in Transgenic Tobacco

#### 2.3.1. Obtainment of Transgenic Tobacco Expressing CAT3 or Its Fusion Form

As mentioned above, CAT3 could enlarge its promotion on the heat tolerance of *E. coli* and yeast via fusion expression engendering enhanced solubility and thermostability. Inspired by this finding, we further conducted verification in plants. We constructed a series of pBI121-derived plant expression vectors of CAT3 and its fusion forms (r/t/p/uCAT3) (Supplementary Figure S3) and generated corresponding transgenic tobacco lines (termed CAT3, r/t/p/uCAT3) by Agrobacterium infiltration.

These transgenic tobacco lines were then examined for target gene expression at the transcriptional level in the main tissues (root, stem, and leaf) by RT-PCR, using WT tobacco as the control. The expression of the internal gene *18S rRNA* was ubiquitous and taken as a reference to rectify the expression level of various CAT3 transgenes. Therein, no distinct difference was found among the main tissues and within the diverse lines of transgenic tobacco (Supplementary Figure S4), indicating a universal expression of various CAT3 transgenes governed by the constitutive *CaMV* 35S promoter.

#### 2.3.2. Fusion Expression Improves the Recovery Growth of CAT3-Expressing Transgenic Tobacco Plants After Heat Stress

**Figure 4:**
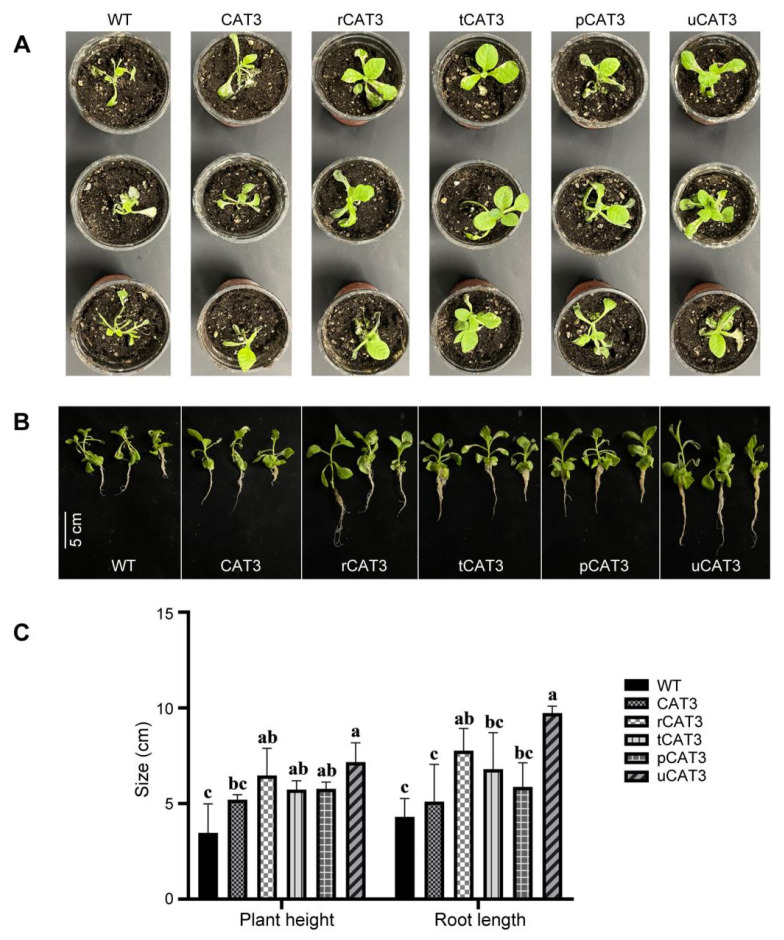
Growth status comparison of WT and various CAT3 transgenic tobacco plants after one-week recovery from heat stress of 12 h at 45 °C. (**A**) The stressed/recovered tobacco plants in soil in pots. (**B**) Full plant morphology. (**C**) Plant height and root length. Variance significance (a, b, c) was defined according to the Duncan test method.

Thirty-day-old WT and transgenic tobacco plants were heat-treated for 12 h at 45 °C, followed by recovery growth for one week. The WT tobacco suffered severe wilting and leaf whitening and even death. In contrast, the transgenic tobacco overexpressing CAT3 protein (i.e., CAT3 tobacco) showed a moderate recovery, but its leaf blanching remained obvious. The transgenic tobaccos overexpressing fusion proteins rCAT3, tCAT3, pCAT3, and uCAT3 (i.e., r/t/p/uCAT3 tobacco) only exhibited weak symptoms of leaf whitening, and their recovery growth was significantly better and faster than that of CAT3 tobacco, with leaves almost fully expanded (Figure 4A). These plants were put together in an array for whole picture comparison, again showing various CAT3 fusion tobaccos as more competent in recovery growth (Figure 4B). Additionally, the plant height and root length in CAT3 tobacco were 1.5 and 1.2 times those of WT tobacco, especially in various transgenic tobaccos expressing CAT3 fusions (especially rCAT3, uCAT3) (Figure 4C). Therefore, in terms of the growth status after heat stress, the transgenic tobacco plants expressing any of the CAT3 fusions outperformed the native form, demonstrating a faster recovery rate and stronger heat tolerance.

#### 2.3.3. Fusion Expression Improves the Seed Germination and Seedling Growth of CAT3-Expressing Transgenic Tobacco After Heat Stress

Under normal conditions (CK), no meaningful difference was found in the seed germination rate (>90%) among WT and all types of CAT3 transgenic tobaccos. In contrast, a parallel test for seeds preheated at 45 °C for 12 h showed a remarkable variance in germination. Therein, WT tobacco seeds hardly germinated, CAT3 tobacco could retain a seed germination rate of approximately 20%, while this maintenance was much higher in transgenic tobaccos with various fusion expressions of CAT3, i.e., germination rates of 42%, 53%, 38%, and 48% for rCAT3, tCAT3, pCAT3, and uCAT3 tobacco, respectively (Supplementary Figure S5).

**Figure 5:**
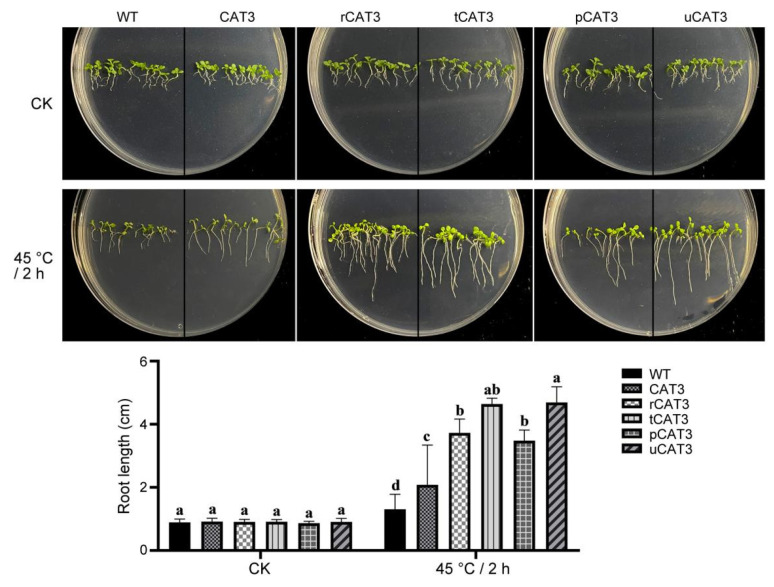
Seedling growth comparison of WT and various CAT3 transgenic tobaccos after heat stress of 2 h at 45 °C. CK: before heat treatment. Variance significance (a, b, c, d) was defined according to the Duncan test method.

We also investigated the seedling growth of various CAT3 transgenic tobaccos recovered from a moderate heat treatment of 2 h at 45 °C. All types of tobacco seedlings had a similar growth status with comparable root length before heat treatment (CK), but the resumed seedling growth after heat stress was differentiated greatly in WT and transgenic tobaccos, i.e., almost ceased in WT tobacco, relatively better in CAT3 tobacco, and the best in various CAT3 fusion (r/t/p/uCAT3) tobaccos. The seedling root length of these transgenic lines sequentially reached 1.5, 2.9, 3.9, 3.4, and 3.9 times that of WT tobacco (Figure 5).

We further examined the survival of tobacco seedlings with one-week recovery after a prolonged heat stress of 6 h at 45 °C. All WT tobacco seedlings died, and only a small number (about 8%) of CAT3 tobacco seedlings resumed growth, whilst the seedling survival rates of transgenic tobaccos with fusion expressions of rCAT3, tCAT3, pCAT3, and uCAT3 were 25%, 22%, 12%, and 25%, respectively (Supplementary Figure S6).

Collectively, these results indicate the introduction of CAT3 in tobacco is beneficial to seed germination, survival, and resumed growth of seedlings after heat stress, and this rise in tobacco thermotolerance can be prominently intensified by fusion expression with effectual partners (e.g., ra3t, tua2, rub).

#### 2.3.4. Fusion Expression Enhances the Ability of CAT3 to Prevent Chlorophyll Destruction and Membrane Lipid Peroxidation from Heat Stress in Transgenic Tobacco Plants

**Figure 6:**
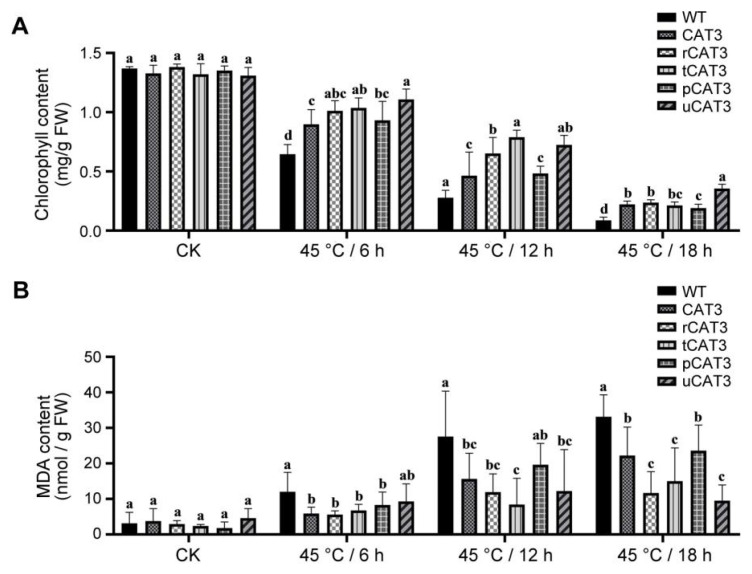
Content comparison of the total chlorophyll (**A**) and MDA (**B**) in the leaves of WT and various CAT3 transgenic tobaccos after a set of heat stresses at 45 °C. CK: before heat treatment. Variance significance (a, b, c, d) was defined according to the Duncan test method.

The main photosynthesis pigment, chlorophyll, is tightly correlated with diverse stresses in plants, and its content fluctuation can be used for evaluating the extent of damage. Without heat stress (CK), the total chlorophyll content was similar in WT and all transgenic tobacco leaves. After a heat treatment for 6 h at 45 °C, it decreased by 44.5% in WT, but notably less so in transgenic tobaccos expressing CAT3 and its various fusions (r/t/p/uCAT3) by 21.5%, 27.6%, 16.0%, 31.1%, and 15.3%. When prolonging the heat stress for 12 h, a greater decrease of chlorophyll content occurred dramatically by 76.2% in WT and moderately in CAT3 and its fusion (r/t/p/uCAT3) transgenic tobaccos (by 59.4%, 53.3%, 36.1%, 64.3%, and 44.7%, respectively). Overall, CAT3 expression protected tobacco chlorophyll from heat destruction, and this role was mostly fortified by fusion expression, especially pronounced with the fusion partners ra3t, tua2, and rub. Nevertheless, this protection became ineffective with further elongation of heat stress up to 18 h, in which the remnants of total chlorophyll reached less than 30% even in uCAT3 (seemingly the best fusion) tobacco (Figure 6A).

Malondialdehyde (MDA) is the final product of lipid peroxidation usually caused by the oxidative stress arising from numerous adverse conditions including high temperature. The accumulation of MDA is a key physiological cue to reflect the damage of membrane lipids and canonically used to appraise the stress tolerance of plants. Before heat treatment (CK), the MDA contents in the leaves of various tobaccos were nearly at a baseline, but gradually elevated during the period of a heat stress at 45 °C. At 6 h, it increased drastically by 3.8 times compared to CK in WT tobacco but only by about 2 times in most of the transgenic lines. At both 12 h and 18 h, the profiles of increasingly produced MDA were similar and demonstrated that the accumulation was significantly lower in all transgenic lines as compared to WT tobacco, especially in CAT3 fusion (except for pCAT3) tobaccos. At 18 h of heat treatment, the MDA content increased by 10.5 times that of CK in WT tobacco and 7.1 times in CAT3 tobacco but only 3.7, 4.8, and 3.0 times in rCAT3, tCAT3, and uCAT3 tobaccos, respectively (Figure 6B). These results clearly indicate that the heterologously introduced CAT3 in tobacco can alleviate the heat-elicited oxidative stress and consequently protect cellular membranes from lipid peroxidation, and this impact is unsurprisingly deepened by fusion expression with effectual partners such as ra3t, tua2, and rub.

#### 2.3.5. Lower H_2_O_2_ Level and Less Cell Damage Under Heat Stress Exist in the Leaves of Transgenic Tobacco with CAT3 Fusion Expression

**Figure 7:**
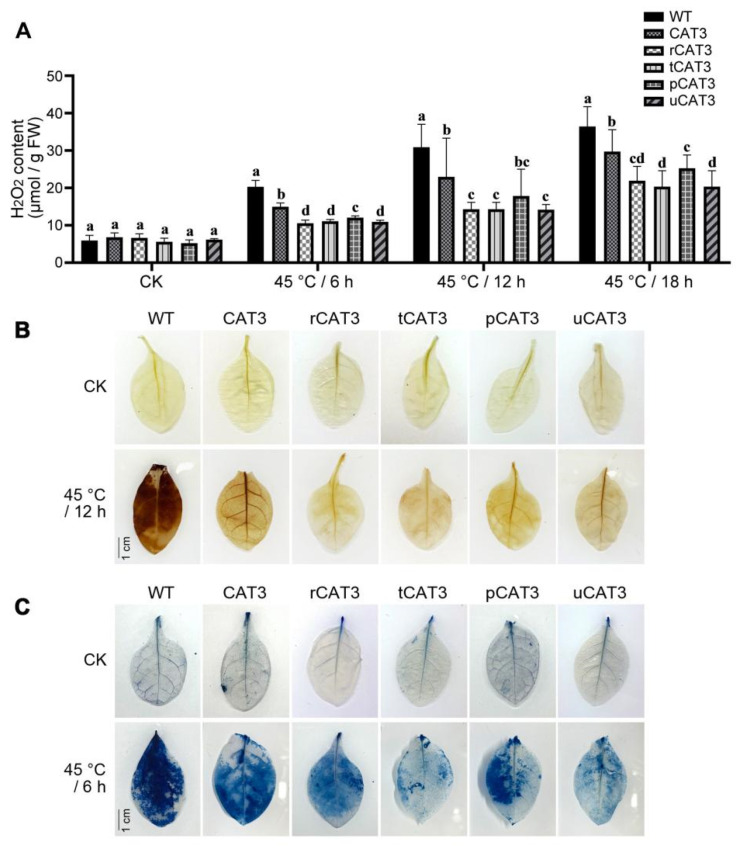
Comparison of H_2_O_2_ accumulation and cell damage in the leaves of WT and various CAT3 transgenic tobaccos after heat stresses at 45 °C. (**A**) Quantitative measurement of H_2_O_2_. (**B**) DAB staining to visualize H_2_O_2_. (**C**) Evans blue staining to visualize cell damage. CK: before heat treatment. Variance significance (a, b, c, d) was defined according to the Duncan test method.

Excess H_2_O_2_ induced by environmental stresses is cell-toxic and scavenged mainly by peroxisomal catalases in plants. Herein, the level of H_2_O_2_ showed no obvious difference before heat treatment (CK) but continuously and differentially increased with the duration of heat stress at 45 °C in the leaves of WT and transgenic tobaccos. At 6 h, the H_2_O_2_ content in WT tobacco increased sharply by 3.4 times compared to CK but moderately in CAT3 tobacco (by 2.2 times) and relatively little in most of the CAT3 fusion (r/t/p/uCAT3) tobaccos (by 1.6, 1.9, 2.3, and 1.8 times, respectively). At 12 h, the H_2_O_2_ level in WT and CAT3 tobaccos continued to rise (up to 5.2 and 3.4 times, respectively) but was augmented slowly in various CAT3 fusion (/r/t/p/uCAT3) tobaccos (2.1, 2.5, 2.7, and 2.8 times, respectively), and this change seemingly lasted up to 18 h of the heat treatment (Figure 7A).

To verify the above H_2_O_2_ measurement, we conducted a routinely used diaminobenzidine (DAB) staining to directly visualize the distribution and the level of H_2_O_2_ in tobacco leaves. In the presence of H_2_O_2_, DAB can be oxidized in cells to produce insoluble brown precipitate, the color intensity of which is proportional to the in situ accumulation of H_2_O_2_. As shown in Figure 7B, before heat treatment (CK), WT and all transgenic tobacco leaves hardly showed brown stains (except for slight staining on the veins), implying scarce background H_2_O_2_ inside. Contrastively, under a heat treatment at 45 °C for 12 h, the leaves of all CAT3-expressing tobaccos displayed fewer brown spots and diffuse staining (i.e., less H_2_O_2_ accumulation) as compared to WT plants, especially in r/t/uCAT3 transgenic lines featuring fusion expression with the effectual partners ra3t, tua2, and rub.

Evans blue staining is often used to detect lack of cell membrane integrity and cell death caused by oxidative stresses. The color intensity of the formed blue precipitate of dye–protein complexes indicates the extent of cell membrane damage and vitality loss. Herein, no obvious blue stains were seen on the leaves of WT and all transgenic tobaccos (except for slight staining on the veins) without heat stress (CK). In contrast, after 6 h of heat treatment at 45 °C, deep blue staining appeared on the leaf in WT tobacco, to a relatively moderate degree in CAT3 tobacco, and even less so in various CAT3 fusion (especially tCAT3, uCAT3) tobaccos (Figure 7C).

Taken together, the above results suggest that the heterologous expression of CAT3 fusion rather compared to its native form may confer a higher potential to counteract the excess of H_2_O_2_ induced by heat stress, leading to less damage to tobacco leaf cells.

#### 2.3.6. Fusion Expression with CAT3 Provides Enhanced Protection for Tobacco Roots Under Heat Stress

**Figure 8:**
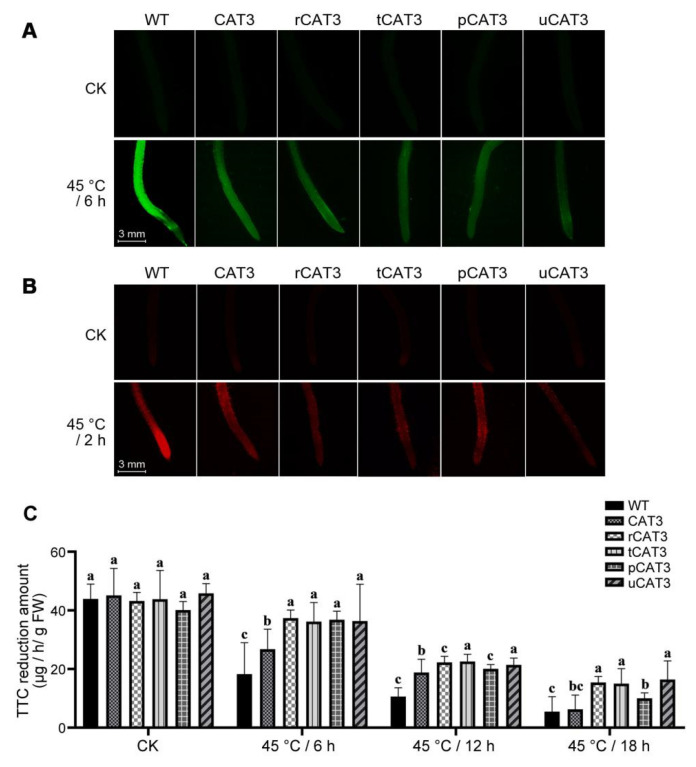
Comparison of ROS accumulation, cell damage, and cell vigor in the roots of WT and various CAT3 transgenic tobaccos after heat stresses at 45 °C. (**A**) H_2_DCFDA fluorescent staining to visualize the ROS in seedling roots. (**B**) PI fluorescent staining to visualize the cell damage in seedling roots. (**C**) TTC reduction to determine the cell vigor in plant roots. CK: before heat treatment. Variance significance (a, b, c) was defined according to the Duncan test method.

We also examined the ROS fluctuation in the root of transgenic tobacco under heat stress. Dichlorofluorescein diacetate (H_2_DCFDA) is a sensitive but non-specific fluorescent probe widely used to monitor the total cellular ROS including H_2_O_2_ in plant tissues, especially suitable for roots. The fluorescence intensity of its oxidized product (2′,7′-dichlorofluorescein, DCF) is positively correlated to the level of ROS in cells. Without heat treatment (CK), only a slight green fluorescence was commonly observed on the roots of seedlings in all types of tobacco, indicating their low background ROS levels. However, after 6 h of heat treatment at 45 °C, the green fluorescence on seedling roots appeared brilliant in WT tobacco but relatively weaker in all transgenic lines, especially in CAT3 fusion (except for pCAT3) tobaccos, indicating that fusion expression can provide CAT3 with a higher efficacy to eliminate ROS in tobacco roots (Figure 8A).

In addition, we investigated the heat-induced cell damage in the root of transgenic tobacco. Propidium iodide (PI) fluorescent staining is routinely used to detect cell membrane integrity and apoptosis and the degree of excited orange fluorescence is proportional to the extent of cell damage. Herein, a scarce baseline fluorescence (i.e., nearly no cell lesions) was commonly found in the roots of both WT and transgenic tobacco seedlings without heat treatment (CK). In contrast, after a heat treatment at 45 °C for only 2 h, the orange fluorescence spread brightly on the seedling root (especially close to the tip) in WT tobacco but seemed relatively mild in CAT3 tobacco and weaker in various CAT3 fusion (except for pCAT3) tobaccos (Figure 8B). This indicates that fusion expression can provide CAT3 with a better protective effect on tobacco roots, with less cell damage and death under heat stress.

This protection was further substantiated by the detection of triphenyl tetrazolium chloride (TTC) reduction (equivalent to cell vigor) in roots. Root vitality is well recognized as an important physiological parameter to reflect plant adaptation to numerous abiotic stresses and negatively correlated with the degree of root damage. As observed from TTC reduction in the roots, WT and all transgenic tobacco plants showed a similar root vitality before heat treatment (CK). However, the root vigor decreased during the heat treatment at 45 °C. At 6 h, the root vitality significantly dropped by 58.4% and 50.2%, respectively, in WT and CAT3 tobacco but was maintained at a high level in various CAT3 fusion (r/t/p/uCAT3) tobacco varieties (with a decrease of less than 20%). This alteration pattern seemingly lasted for longer periods (12 h, 18 h) of the heat treatment, along with increasing loss of root vigor. At 12 h, only a quarter of the root vitality remained in WT tobacco but nearly half remained in various CAT3 transgenic tobaccos, especially those hallmarked by fusion expression. At 18 h, most of CAT3 fusion (r/t/uCAT3) tobaccos still showed a moderate root vigor (above 35% remained), while it became almost indistinguishable (nearly 15% left) between WT tobacco and the transgenic line expressing non-fused CAT3 (Figure 8C). Overall, it can be concluded that CAT3 provides some protection for the roots from vigor loss when heterologously expressed in tobacco, and its diverse fusion forms apparently perform much better, especially with the fusion partners ra3t, tua2, and rub.

#### 2.3.7. Enhanced Thermotolerance in Tobacco Is Solely Ascribed to the Catalase Activity of Introduced CAT3 or Its Fusion Form

**Figure 9:**
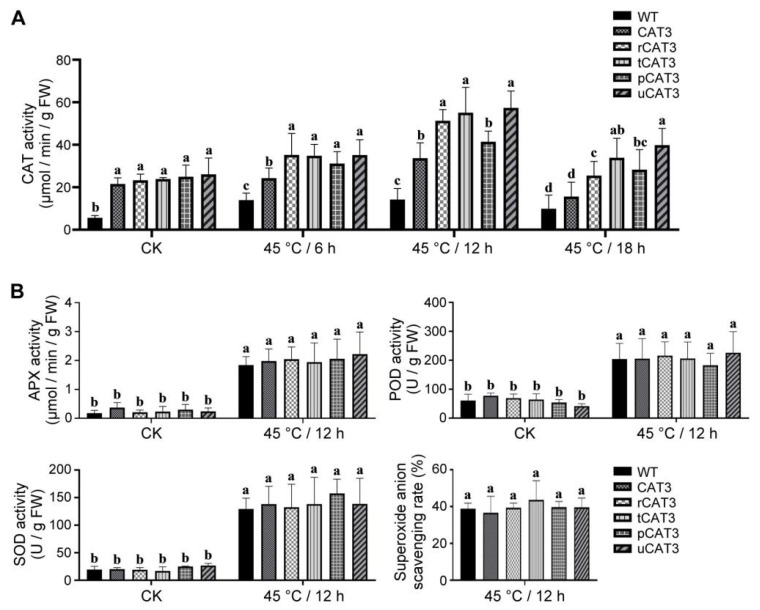
Comparison of activities of the main antioxidative enzymes and superoxide-anion-scavenging rate in the leaves of WT and various CAT3 transgenic tobaccos after heat stresses at 45 °C. (**A**) CAT activity. (**B**) APX, POD, SOD activities and superoxide-anion-scavenging rate. CK: before heat treatment. Variance significance (a, b, c, d) was defined according to the Duncan test method.

As described above, CAT3 tobacco showed an improved thermotolerance compared to WT plants under heat stress, with less accumulation of H_2_O_2_, total ROS, and MDA, weaker destruction of chlorophyll and membrane integrity, and less damage to leaf and root cells. Most noteworthily, this effect could be significantly promoted by fusion expression with selected partners. The introduced CAT3 and its fusion forms were assumed to be the primary contributors and therefore examined for their catalase activities in the leaves of transgenic tobacco. Without heat treatment (CK), all transgenic lines similarly exhibited a remarkably higher (about 4-fold) CAT enzymatic activity compared to WT tobacco, indicating that heterologous overexpression of CAT3 and its various fusion forms can greatly elevate the total CAT activity in tobacco. Under a heat treatment at 45 °C, the total CAT activity varied with stress time and was differentiated in various types of tobacco. At 6 h, the increase in CAT activity reached up to 2.5-fold of CK in WT tobacco and also remarkably occurred in CAT3 and its fusion (r/t/p/uCAT3) tobaccos by 1.74, 2.5, 2.4, 2.2, and 2.5 times that of WT, respectively. At 12 h, the CAT activity further increased, only slightly in WT tobacco (about 3-fold that of CK) but still notably in most of the transgenic lines (2.3, 3.6, 3.9, 2.9, and 4.0 times that of WT, respectively). At both time-points, rCAT3, tCAT3, and uCAT3 tobaccos showed significantly higher CAT activity as compared to the transgenic line expressing native CAT3. However, when the treatment time reached 18 h, the CAT enzymatic activity in all types of tobacco decreased, overall resembling the scenario at 6 h with a small drop. Nevertheless, it become similarly low in WT and CAT3 tobaccos yet still maintained at a relatively high level in transgenic lines expressing various CAT3 fusions (Figure 9A). These dynamic changes in total CAT activity in transgenic lines during the full period of heat stress are speculated to include the heat-responsive expression of endogenous catalases that existed in WT tobacco (a pattern of continuous increase at 6 h and 12 h but a decrease at 18 h). Overall, during heat stress, CAT3 tobacco exhibited a higher CAT activity than WT plants, which was further surpassed by transgenic lines expressing various CAT3 fusions (especially rCAT3, tCAT3, and uCAT3). Meanwhile, we checked the activities of the other main antioxidative enzymes (e.g., ascorbate peroxidase (APX), peroxidase (POD), and SOD) involved in plant ROS control and redox homeostasis. As shown in Figure 9B, in the leaves of WT and all transgenic tobaccos, the activities of these endogenous enzymes had no obvious difference before heat treatment (CK). This was also the case after a heat treatment of 12 h at 45 °C, although there was a dramatic increase in their activity due to their heat-responsive expression. Moreover, the superoxide anion radical (O_2_^−^)-scavenging rate in leaves also seemed to be identical in WT and transgenic tobaccos, matching their similar SOD activities. The results of these internal antioxidative factors can suggest that the thermotolerance improvement and corresponding physiological variances in transgenic lines as aforementioned are only ascribed to the heterologous introduction of various CAT3 forms.

#### 2.3.8. Fusion Variants of CAT3 Are More Thermostable than Its Native Form in Transgenic Tobaccos Under Heat Stress

**Figure 10:**
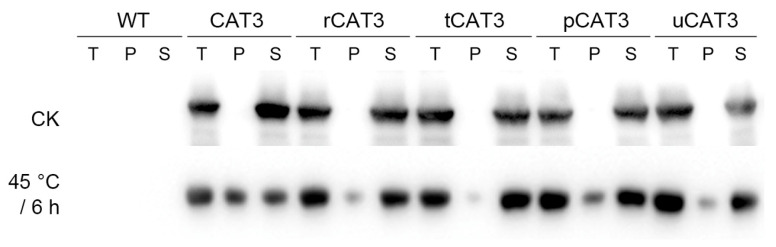
Immunoblotting detection of the solubility changes in CAT3 and its fusion forms (r/t/p/uCAT3) expressed in transgenic tobaccos after heat stress (45 °C/6 h). CK: before heat treatment; T: total proteins in an aliquot of the homogenates of tobacco leaves. S, P: the supernatant and pellet fraction of ‘T’ by centrifugation, respectively.

Since the tobacco thermotolerance improvements mentioned above are only related to the introduced CAT3 or its fusion form, their effect variance should correspond to their different protein thermostabilities, i.e., the ratio of soluble/active remnants after heat stress. Therefore, we examined the solubility changes in various CAT3 proteins expressed in transgenic tobacco plants after a heat treatment of 6 h at 45 °C by immunoblotting with the myc-tag antibody (various CAT3 proteins contain an epitope of myc-tag at the N-terminal). We found that all forms of CAT3 heterologously expressed in tobacco were almost in the soluble fraction before heat treatment (CK) but emerged after heat stress as aggregates to differential extents, i.e., non-fused CAT3 (nearly half), pCAT3 (about a quarter), rCAT3 and uCAT3 (about one-eighth), tCAT3 (scarce) (Figure 10). These results indicate that the fusion variants of CAT3 (especially with the partners tua2, ra3t, rub) in transgenic tobaccos can generally maintain a higher thermostability and a larger amount of soluble/active form to eliminate deleterious H_2_O_2_ under heat stress and thus imbue plants with a more enhanced thermotolerance, as compared to its native form.

## 3. Discussion

### 3.1. Fusion Expression Improves the Thermostability of Arabidopsis CAT3 with Better Activity Maintenance Under Heat and Enlarges CAT3-Mediated Thermotolerance in Transgenic Organisms (E. coli, Yeast, and Tobacco)

Climate change, especially global warming, has become a serious threat to crop productivity [45]. Continuous high temperatures can cause ROS imbalance and oxidative stress, resulting in severe physiological damage and irreversible inhibition of plant growth and development [15]. Therefore, controlling the accumulation of ROS is of critical importance in developing plant heat tolerance, in which CAT, as a major H_2_O_2_ scavenger, is certainly involved. However, plant CAT proteins are likely intrinsically heat labile and prone to aggregation under high temperature, which might compromise their enzymatic activity [26,27] and consequently impede plant thermotolerance. Therefore, our presented work focused on how to beneficially engineer the thermostability of CAT enzymes to enhance plant heat tolerance.

We first verified Arabidopsis CAT3 was of poor solubility when heterologously expressed in *E. coli* even under low temperature, and the recombinant CAT3 unsurprisingly had poor thermostability. When attaching our selected fusion partners (ra3t, tua2, p60c, rub) at the N-terminal, all CAT3 fusion proteins demonstrated enhanced solubility and thermostability (Figure 1 and Supplementary Figure S1). These fusion moieties could also remarkably protect CAT3 from heat inactivation (Figure 2). In addition, the recombinant *E. coli* and yeast strains harboring the constructs of CAT3 fusions commonly outperformed those with non-fused CAT3, exhibiting a much better thermotolerance with higher survivorship at high temperatures (Figure 3). This effect may have arisen from the improved thermostability and activity maintenance under heat in CAT3 fusion variants (Figure 1 and Figure 2 and Supplementary Figure S2).

Collectively, these results confirmed the efficacy of these solubilizing/thermostabilizing fusion partners on other client proteins and reinforced the notion that elevated solubility can contribute to protein thermostability [35,37,38,39]. Thermostable protein should be generally in a soluble/active form, but solubility cannot always guarantee activity and thermostability for proteins. Only solubilized proteins with good activity maintenance under heat can be regarded as the truly thermostabilized. Fortunately, our CAT3 fusion variants fit this aspect.

Moreover, the effects of fusion expression on CAT3 were evaluated in plants. Heterologous overexpression of CAT3 improved the heat tolerance of tobacco, which could be further potentiated when introducing any of our selected solubilizing/thermostabilizing fusion partners, as evidenced by numerous phenotypic and physiological analyses (Figure 4, Figure 5, Figure 6, Figure 7, Figure 8 and Figure 9 and Supplementary Figures S5 and S6). Comparatively, transgenic tobacco plants expressing CAT3 fusions had much faster growth recovery and higher CAT activities after heat stress, with lower accumulation of H_2_O_2_, total ROS, and MDA. The advantages of fusion expression were also noticeable in terms of seed germination rate, seedling survival rate, root vigor, and mitigation of leaf/root cell damage under high-temperature conditions. Meanwhile, various CAT3 fusion transgenic plants retained much higher chlorophyll contents, indicating that fusion expression effectively enhances the activity of CAT in tobacco and reduces the damage of H_2_O_2_ and other ROS to photosynthetic pigments. This result directly mirrored the amelioration of poor plant heat tolerance with typical characters of leaf chlorosis and photosynthetic rate decay [27]. Additionally, determination of the activities of the other main antioxidative enzymes (e.g., APX, POD, and SOD) and superoxide-anion-scavenging rate revealed that the fortification of CAT3-mediated tobacco thermotolerance was only ascribed to the fusion expression with those effectual tags that provide thermostable and activity-stable fusion variants of CAT3 in transgenic tobaccos (Figure 10).

### 3.2. HAMP Fusion Tags Demonstrate a Common Thermostabilizing Effect That Is, However, Not Parallel to Their Acidity

Among the used fusion partners, all three HAMPs (ra3t, tua2, p60c) were consistently effectual on CAT3. Besides the potential molecular chaperone activity [44], these fusion tags can massively endow CAT3 protein with negative charge (Table 1), thus forming a stronger charge repulsion force that makes protein molecules struggle to aggregate with an improved solubility [37,38,39,43]. At high temperatures, this molecular repulsion still exists and can enable CAT3 to maintain a relatively high solubility, thereby passively increasing its thermostability to resist heat inactivation and confer its recombinant organisms (*E. coli*, yeast, tobacco plant) an enhanced heat tolerance. However, this effect does not seem to be proportional to the acidity of HAMPs (ra3t, tua2, p60c). Overall, ra3t and tua2 are of similar efficacy, resembling our previous results [37,38,39]. In contrast, p60c performed relatively weakly, despite having the highest acidity (with net negative charges more than twice that of ra3t and tua2, Table 1). This discrepancy can be explained by certain special amino acid residues and their distribution within numerous acidic amino acids being determinants of HAMP effectiveness.

### 3.3. Unique Features Imbue P. furiosus Rubredoxin with a Thermostabilizing Efficacy Similar to That of HAMP Tags

In this study, we also utilized the rubredoxin (rub) from the hyperthermophilic archaea *P. furiosus* as a fusion moiety to improve CAT3 thermostability and its related roles and achieved an efficacy comparable to or even slightly better than that of HAMP tags (ra3t, tua2). Proteins in hyperthermophiles generally have high thermal stability [31], as represented by *P. furiosus* rub despite its small size [41]. This ultra-thermostable rub with an Fe-S center has been used as a fusion partner to visually monitor the purification process of target proteins [42] and also improve the solubility/thermostability/activity of several target proteins and the heat tolerance of their recombinant microbes in our previous study [36]. Herein, the solubilizing/thermostabilizing effect of this rub tag was again validated on the heat-labile enzyme protein CAT3, which could additionally imbue transgenic tobacco plants with an improved heat tolerance. Speculatively, *P. furiosus* rub, as a fusion partner, might in cis convey its ultra-high thermal stability on its linearly linked target proteins via enthalpic and entropic mechanisms, just like other proteins derived from thermophilic microorganisms [31,32,33,34]. Moreover, rub also has a high degree of acidity (Table 1), probably moonlighting a similar role to the HAMP tag.

In addition, rubredoxin can directly participate in the electron transfer in peroxidase reactions [46]. Thus, it has been regarded as a key component of a novel non-heme antioxidation protection system that can complement the classical oxidative stress defense system relying on CAT/APX (heme-containing) and SOD [47,48]. Their synergistic effects cannot be ruled out. In some microorganisms, rubredoxin naturally fuses with a few enzymes to form complexes with higher catalytic activity, wherein it can channel electrons faster and more efficiently [49,50]. Rubredoxin or its analogous proteins also exist in plants with certain functions [51]. Rubredoxin may have a molecular chaperone activity, helping other proteins assemble or fold correctly. For example, in *Chlamydomonas reinhardtii*, thylakoid membrane-bound rubredoxin 1 (RBD1) acts as a photosystem II (PSII) assembly factor, containing a redox-active rubredoxin domain (exposed on the stromal side, involved in PSII repair) and a single C-terminal transmembrane α-helix (TMH) domain (essential for de novo PSII assembly) [52]. RBD1 can also promote the proper folding of the D1 component, possibly via delivery or reduction of the non-heme iron during PSII assembly. The double (RBD1, FtsH1 protease)-deficient mutant shows the accumulation of a 23 kDa truncated D1 protein (a marker typically associated with structural changes resulting from photodamage of PSII) under dark and low-light conditions [53]. Moreover, rubredoxin or its analogues may be involved in the responses of plants to abiotic stresses, maintaining normal electron transfer and related metabolisms and reducing ROS accumulation under stress environments [54,55]. Its overexpression enhances the tolerance of transgenic plants to various stresses such as salt and alkali [55].

Collectively, rubredoxin likely possesses many characteristics suitable for fusion tags (e.g., small size with minimal disturbance to target proteins, high stability, a certain amount of acidity, chaperoning activity, and even native existence as a fusion-bound electron carrier), besides antioxidation properties, a natural linkage to CAT, and a direct role in stress tolerance. Therefore, it is quite understandable that the fusion with *P. furiosus* rub can improve CAT3 thermostability and activity, showing a consistently remarkable enhancement on heat tolerance in bacteria, yeast, and tobacco plants.

### 3.4. Fusion Expression May Promote CAT-Mediated Tolerance to Other Environmental Stresses Beyond Heat

Besides heat destruction, proteins also tend to aggregate due to intracellular molecular crowding under osmotic stresses such as drought and salt [56,57]. CAT has a universal role in plant response and adaptation to diverse abiotic stresses including heat, drought, and salt [10,11,12,13,14,15,16]. However, it necessitates accessory factors for folding, oligomerization, antiaggregation, stabilization, and activation [17,18,19,23,24,25,26], of which TaWD40-4B.1 is particularly associated with CAT-mediated drought tolerance [24]. Accordingly, our constructed fusion variants of CAT3 (with enhanced solubility, especially by charge repulsion force via HAMP tags) might have the potential to antagonize aggregation induced by molecular crowding, thus applicable for improving plant tolerance to osmotic stresses beyond heat. Additionally, CAT is involved in plant photorespiration [6], pexophagy [8], immunity [9], and other ROS-dominated developmental processes [7], which might also be ameliorated by these potentiated fusion variants of CAT3, especially under heat stress conditions.

### 3.5. Fusion Expression-Directed Protein Engineering Can Be Potentially Applicable for Developing Climate-Resilient Crops in Future Agriculture

As seen from abundant evidence (obtained herein and previously in [31,32,33,34,35,36,37,38,39]) and compared with mutagenesis-based approaches [28,29,30], the fusion expression strategy used for protein stability engineering is simple for molecular manipulation, universally applicable with minimal side-effects, and has considerable efficacy, just depending on the addition of effectual fusion partners at the proper positions (normally N- or C-terminus) of any target proteins without changes in amino acid sequence. Recently, several solubilizing/thermostabilizing tags (e.g., NEXT [35], rub [36], HAMPs [37,38,39]) have been repeatedly verified, and new candidates are forthcoming. Prospectively, this strategy and these effectual tags can be further exploited to improve the thermostability of other proteins (e.g., Rubisco activase in photosystem [58], plastid translation elongation factor EF-Tu [59]) that are crucial in plant biology but inherently heat labile or aggregation prone, thus ameliorating plant tolerance to abiotic stresses (especially high temperature). Furthermore, this protein engineering strategy can be combined with genome editing biotechniques for plant trait improvements, since short fusion tags are also suitable for genomic insertion via current gene editor tools [60]. Therefore, the application potential of the protein fusion expression strategy is not negligible in the field of plant biotechnology for sustainable agriculture, especially when developing climate-resilient crops [61].

## 4. Materials and Methods

### 4.1. Vector Construction

The primers used in this study are listed in Table S1. The *CAT3* gene (At1g20620) was amplified by RT-PCR from Arabidopsis leaf RNA with primer pair AtCAT3-5Nd/AtCAT3-3Xh, then recombinantly ligated into *Nde* I, *Xho* I-digested (Thermo Scientific, Waltham, MA, USA) plasmid pET32a(+) (EMD Biosciences (Novagen), San Diego, CA, USA) by the ClonExpress II Cloning Kit (Vazyme Biotech, Nanjing, China) to create the prokaryotic expression vector pET(CAT3).

The DNA fragments of fusion tags ra3t (r), tua2 (t), and rub (u) were individually re-amplified from plasmids pET(r-J), pET(t-J) [39], and pET(PsPtx-rub) [36], using primer pairs PetNd-uF/rCAT3-R, PetNd-uF/tCAT3-R, and RubNd-F/uCAT3-R. The coding sequence of fusion tag p60c (p) (i.e., C-terminal hyperacidic extension of *Plasmodium berghei* ANKA chaperonin cpn60, XP_677199) was synthesized by two sequential rounds of overlapped PCR using primer pairs P60c-Fin/P60c-Rin and P60c-Fou/P60c-Rou. These amplicons of fusion tags were then recombinantly inserted into *Nde* I-linearized plasmid pET(CAT3) to generate a set of prokaryotic fusion expression vectors, pET(rCAT3), pET(tCAT3), pET(pCAT3), and pET(uCAT3), respectively.

Using the full set of the above *CAT3* prokaryotic vectors as templates, the DNA fragments of CAT3 and its various fusion forms were individually re-amplified with the forward primer (CAT3y-F, rCAT3y-F, tCAT3y-F, pCAT3y-F, or uCAT3y-F) and the common reverse primer CAT3y-R, then recombinantly cloned into dually *Kpn* I, *Xba* I-digested (Thermo Scientific, Waltham, MA, USA) plasmid pYES2 (Invitrogen, Carlsbad, CA, USA) to construct the corresponding set of yeast expression vectors, pYES2(CAT3), pYES2(rCAT3), pYES2(tCAT3), pYES2(pCAT3), and pYES2(uCAT3), respectively.

Likewise, using the serial *CAT3* prokaryotic vectors as templates, the DNA fragments of CAT3 and its various fusion forms were individually re-amplified with the forward primer (CAT3-5F, rCAT3-5F, tCAT3-5F, pCAT3-5F, or uCAT3-5F) and the common reverse primer PetHis-dRv. These amplified products were then used as the templates for a subsequent round of PCR with the common primer pair VecEn-5Xb, CAT3-3Xp. The final amplicons were recombinantly ligated into a modified pBI121 plasmid (Clontech, Mountain View, CA, USA) dually digested by *Xho* I, *Xba* I (Thermo Scientific, Waltham, MA, USA) to generate the corresponding set of plant expression vectors, pBI(CAT3), pBI(rCAT3), pBI(tCAT3), pBI(pCAT3), and pBI(uCAT3), respectively.

### 4.2. Recombinant Protein Expression in E. coli

*E. coli* strain BL21(DE3) containing each *CAT3* prokaryotic expression vector was grown in LB medium (+100 µg/mL ampicillin) at 37 °C to an OD_600_ of 0.6, then induced overnight at 20 °C by a final concentration of 0.2 mM isopropyl β-D-thiogalactoside (IPTG) (Sigma-Aldrich, St. Louis, MO, USA). After induction, 14 mL of bacterial cells was collected by centrifugation and lysed in 4 mL of 100 mM PBS buffer (pH 7.4) by ultrasonification. Each aliquot (16 µL) of crude cell lysate (T) was fractionated by centrifugation into the supernatant (S) and the pellet (P) which was immediately dissolved in the same volume of PBS buffer. Meanwhile, the precipitate of 200 µL bacterial cells before induction was resuspended in 16 µL PBS buffer as the uninduced sample (UI). These samples were subsequently subjected to 12% SDS-PAGE with Coomassie blue staining.

### 4.3. Thermostability and Activity Analysis of E. coli-Expressed Recombinant CAT3 Proteins

The induced *E. coli* expression of each *CAT3* prokaryotic vector was conducted as mentioned above. Aliquots (each 16 µL) of the supernatant of bacterial cell lysates were heat-treated with a gradient temperature (25–45 °C, 5 °C interval) for 30 min, and then each was partitioned into the supernatant (S) and pellet (P) for 12% SDS-PAGE with Coomassie blue staining. The varied densities of recombinant protein bands on gels in supernatant (S) and pellet (P) samples at each heating point (i.e., protein solubility changes with increasing temperature) were recorded for initial judgement of CAT3 thermostability.

Meanwhile, another set of heat-treated samples for each CAT3 recombinant expression were separated on another SDS-PAGE gel and then semi-dry transferred to PVDF membrane (Millipore, Billerica, MA, USA) for immunoblotting. After blocking and washing, the membrane was sequentially reacted with myc-tag mouse monoclonal antibody (AF0033, Beyotime, Shanghai, China) at a dilution of 1:1000 and horseradish peroxidase (HRP)-conjugated goat antimouse IgG (H + L) (A0216, Beyotime, Shanghai, China) as the secondary antibody at a dilution of 1:2000. The antigen–antibody complexes were detected by enhanced chemiluminescence using the BeyoECL Star kit (P0018AS, Beyotime, Shanghai, China). The varied densities of visualized bands on blots in supernatant (S) and pellet (P) samples at each heating point were recorded to appraise CAT3 thermostability again.

In addition, the serial heat-treated samples for each CAT3 expression were directly subjected to total catalase activity measurement by a commercial CAT assay kit (G0105F, Grace Biotech, Suzhou, China), using WT *E. coli* as the baseline reference. Briefly, samples were incubated with excess H_2_O_2_ for decomposition by catalase for 5 min, and the remaining H_2_O_2_ reacted with a substrate by adding peroxidase to generate a red product, N-4-antipyryl-3-chloro-5-sulfonate-p-benzoquinonemonoimine, which absorbs maximally at 510 nm. Catalase activity was thus determined by measuring the decomposed H_2_O_2_ and further normalized against the total protein concentration of the same sample (determined by the BCA method) to obtain the specific activity expressed as U/mg protein.

### 4.4. Survival Test by Dot-Plating of Heat-Treated Recombinant E. coli and Yeast Strains

The recombinant *E. coli* strain BL21(DE3) harboring each *CAT3* serial prokaryotic expression vector or the empty vector pET32a(+) was cultured to OD_600_ = 0.6 in LB medium (+100 µg/mL ampicillin) at 37 °C, then induced for 4 h at 37 °C with 0.5 mM IPTG. Subsequently, all bacterial cells were adjusted to an OD_600_ of 1.4 and heat-treated at 44 °C, 48 °C for 2 h. Finally, 2 µL of each serial dilution (1-, 10-, 100-, 1000-, 10,000-fold) for every treated strain was dot-plated in a row on solid LB medium and incubated at 37 °C for 1–2 d.

Likewise, *S. cerevisiae* strain INVSC1 (Invitrogen, Carlsbad, CA, USA) containing each *CAT3* serial yeast expression vector or the empty vector pYES2 was incubated at 30 °C in YPD medium (1% yeast extract, 2% peptone, 2% dextrose) to an OD_600_ of 0.4, then transferred to YPG medium (1% yeast extract, 2% peptone, 2% galactose) for induction for 24 h at 30 °C. Afterwards, the induced yeast cells were adjusted to an OD_600_ of 2, treated under heat (48 °C, 51 °C) for 2 h, and finally 2 µL of each serial dilution (1-, 10-, 100-, 1000-, 10,000-fold) for every treated strain was dot-plated in a row on solid YPD medium and incubated at 30 °C for 2–4 d.

### 4.5. Tobacco Transformation and Identification of Transgenic Plants

Agrobacterium infiltration of leaf discs was used for tobacco (*Nicotiana tabacum* L. cv *Xanthi*) transformation, according to a standard protocol [62]. Briefly, small pieces (about 1 cm^2^) of aseptic WT tobacco leaves were transiently submerged in the culture of *A. tumefaciens* strain EHA105 harboring each *CAT3* serial plant expression vector and selected on Murashige and Skoog (MS) medium with 100 mg/L kanamycin in a growth chamber in standard conditions (26 °C, 70% humidity, 1600 lux, and a regime of 16 h light/8 h dark). Positive transgenic tobacco plants were identified by multiple genomic PCR with reciprocally combinatorial primer pairs derived from CAT3 inserts and the vector pBI121.

### 4.6. Heterologous Gene Expression in Transgenic Tobacco Analyzed by RT-PCR

Various CAT3 expressions in their transgenic tobaccos were analyzed by RT-PCR. First, total RNA from the main tissues (root, stem, and leaf) of transgenic tobacco plants was extracted by Trizol reagent (Invitrogen, Carlsbad, CA, USA) and then reversely transcribed by the EasyScript^®^ First-Strand cDNA Synthesis SuperMix (TransGen, Beijing, China) as the templates for PCR detection of *CAT3* transcriptional expression with the common primer pair Myc-Fw/CAT3-iRv. By the same means, the expression of an internal reference gene (tobacco *18S rRNA*) was analyzed with its specific PCR primer pair Nt18S-iFw/Nt18S-iRv.

### 4.7. Growth Status Evaluation of Tobacco Seedlings and Plants After Heat Stress

Seeds of WT and transgenic tobaccos were surface-sterilized according to a routine procedure (immersing in 75% alcohol for 1 min and 10% sodium hypochlorite solution for 8 min, washing three times with sterile water, and air-drying on a clean bench). Afterwards, they were evenly sowed on the central horizontal axis of MS medium for vernalization for two days at 4 °C and vertically cultivated in a 26 °C standard growth chamber for one week, then the root lengths of seedlings were measured. Subsequently, the seedlings were heat-treated at 45 °C for 2 h in the dark and returned for 10 d, then their root lengths were again measured. The seedling root lengths at both stages were compared among WT and transgenic tobaccos.

Meanwhile, two-week-old WT and transgenic tobacco plants in soil pots with similar growth statuses were selected for a heat treatment of 12 h at 45 °C and then transferred to normal conditions (26 °C) to resume growth for one week. The full plant morphology was recorded photographically, then the aboveground height and root length of each treated plant were measured.

### 4.8. Evaluation of Tobacco Seed Germination Rate and Seedling Survival Rate After Heat Stress

Surface-sterilized seeds of WT and various transgenic tobaccos after vernalization were evenly and horizontally sown on MS medium for normal germination as the control (CK). Meanwhile, a duplicate set of seeds were heat-treated at 45 °C for 12 h, then returned to standard conditions for germination. Ten days later, the seed germination rates were calculated and compared among WT and transgenic tobaccos.

In addition, another duplicate set of seeds were normally germinated and cultured on MS medium for three weeks. Afterwards, the grown seedlings were heat-treated at 45 °C for six hours in the dark, and then returned for one week. The survival rates of seedlings were determined and compared among WT and transgenic tobaccos.

### 4.9. Physiological Analyses of Tobacco Plants After Heat Stress

WT and transgenic tobacco plants were grown until 30 days old and subjected to heat treatments and then physiological indexes were measured. The contents of total chlorophyll (expressed as mg/g FW) in leaves were spectrometrically determined as described previously [63]. The contents of MDA (nmol/g FW) and H_2_O_2_ (µmol/g FW) in leaves were determined by the methods of thiobarbituric acid [64] and titanium sulfate [65], respectively. Root vigor (µg/h/g FW) was determined by the enzymatic reduction assay of TTC, according to a literature protocol [66]. The activities of the main antioxidative enzymes (CAT, APX, POX, SOD) in leaves were determined using the corresponding kits (G0105F, G0203F, G0107F, G0101F) and instructions from the manufacturer (Grace Biotech, Suzhou, China) and expressed as µmol/min/g Fw (for CAT, APX) and U/g Fw (for POX, SOD).

DAB staining was used to in situ detect H_2_O_2_ in leaves, according to a method described previously [67]. The leaves of 30-day-old tobacco plants after a heat treatment of 12 h at 45 °C were detached and incubated in 1 mg/mL DAB solution (Sigma-Aldrich, St. Louis, MO, USA) for 8 h in the dark at room temperature on a rotary shaker. After a complete rinsing with water, the leaves were immersed in a decolorizing solution (ethanol:glycerol:acetic acid at a ratio of 3:1:1) for 15 min at 95 °C until the background was bleached without native pigments, then photographed after cooling.

Evans blue staining was conducted to examine leaf cell damage as described previously [68]. The detached leaves of 30-day-old tobacco plants after a heat treatment of 6 h at 45 °C were completely submerged in 0.25% (*w*/*v*) Evans blue solution (Sigma-Aldrich, St. Louis, MO, USA) for 24 h at room temperature on a rotary shaker, then rinsed three times to clean the dye debris off the leaf surface. Subsequently, the leaves were incubated in a boiled decolorizing solution for 5–10 min until the background turned white, then photographed.

PI staining was performed to detect root cell damage, according to a method described previously [69]. Two-week-old tobacco seedlings were treated at 45 °C for 2 h, from which the root tips (approximately 1 cm) were cut and immediately immerged into 10 μmol/mL PI solution (Sigma-Aldrich, St. Louis, MO, USA) for 2 min at room temperature. After water-rinsing three times to remove excess dye, the cleaned root tips were placed on a slide glass and covered with a layer of 25% glycerol, the fluorescence from which was detected by an inverted microscope Olympus CKX53 (OLYMPUS, Tokyo, Japan) (535 nm excitation/615 nm emission) and imaged by a coupled CCD camera.

H_2_DCFDA staining was used to detect the total ROS in root cells as described previously [70]. Three-week-old tobacco seedlings were treated at 45 °C for 6 h, the root tips (approximately 1 cm) of which were excised and immersed into 10 μmol/L H_2_DCFDA solution (Sigma-Aldrich, St. Louis, MO, USA) for 1 h at 37 °C in the dark. After rinsing three times with water, the root tips were placed on a slide glass for fluorescence detection and recording with the aforementioned apparatus (495 nm excitation/525 nm emission).

### 4.10. Fractionation of Soluble and Insoluble Proteins from Yeast Cells and Tobacco Plants for Immunoblotting

Yeast culturing and inducible expression were the same as for the above dot-plating test. After adjusting OD_600_ to 2, the induced yeast cells were heat-treated for 2 h at 48 °C in a water bath. Subsequently, 8 mL of each yeast culture (heat-treated and non-treated) was harvested by centrifugation and ultrasonically lysed in 2 mL of 100 mM PBS buffer (pH 7.4). Each aliquot (32 µL, about 15 µg total protein by BCA determination) of crude cell lysate (T) was partitioned by centrifugation into the supernatant (S) and the pellet (P) which was immediately dissolved in PBS buffer of the same volume as ‘T’.

The extraction of plant soluble and insoluble proteins was generally performed in accordance with a previous protocol [27] with minor modifications. Briefly, 0.5 g of leaves of 30-day-old tobacco plants before and after a heat treatment for 6 h at 45 °C was harvested, ground in liquid nitrogen, and homogenized in an extraction buffer (EB) (100 mM Tris-HCl, pH 8.0, 10 mM NaCl, 1 mM EDTA, 1% Triton X-100, 0.2% β-mercaptoethanol, 1x plant protease inhibitor cocktail (Sigma-Aldrich, St. Louis, MO, USA)). The extracts were filtered through miracloth (Millipore, Billerica, MA, USA) and cleared by a low-speed centrifugation (50× *g*, 10 min, 4 °C) to give homogenates (T, total protein). After a high-speed centrifugation (8000× *g*, 40 min, 4 °C), the supernatants (S) from ‘T’ aliquots (32 µL, approximately 50 µg total protein for each ‘T’ by BCA determination) were removed and kept, while the pellets (P) were washed once (resuspended in EB and spun) and finally dissolved in EB of the same volume as ‘T’.

Afterwards, corresponding volumes of 5× loading buffer were added, and equal volumes of the full set of samples (T, P, S) from yeast cultures or plants were subjected to SDS-PAGE and immunoblotting with the myc-tag antibody as described in above for thermostability analysis of recombinant CAT3 proteins.

### 4.11. Bioinformatic and Statistical Analyses

Vector construction and analysis of basic protein features (e.g., size (amino acids, aa), isoelectric point (*p*I), molecular weight (M.W.), and net charge at pH 7.0) were assisted by the software Vector NTI Suite 11.5 (Invitrogen, Carlsbad, CA, USA). All statistics were performed by the Excel 2019 program, using the data (mean ± SD) from three separate experiments. Variance analysis was conducted by the SPSS Statistics 21.0 program (IBM-SPSS, Chicago, IL, USA), with the Duncan test method to define significance (a, b, c, etc.).

## 5. Conclusions

In this study, Arabidopsis CAT3 was validated as having poor thermostability and was then engineered by N-terminal fusion expression in *E. coli* using several solubilizing/thermostabilizing fusion partners (three hyperacidic mini-peptides and the short hyperthermostable rubredoxin from *P. furiosus*). All these fusion moieties were commonly effectual on CAT3 to enhance its solubility and thermostability, protect it from heat inactivation, and magnify its improvement of heat tolerance in *E. coli*, yeast, and most importantly in tobacco. This functional potentiation in CAT3 fusion variants was ascribed to their improved thermostability and activity maintenance under high temperatures. Prospectively, this protein engineering strategy by fusion expression would provide an alternative biotechnical path for ameliorating plant thermotolerance and molecular breeding of climate-resilient crops.

## Figures and Tables

**Table 1:** The properties of fusion partners, target protein CAT3, and their fusion proteins.

Name	Size (aa)	M.W. (kDa)	*p*I	Net Charge at pH 7.0
Fusion partner				
ra3t (r)	37	3.97	3.01	−14
tua2 (t)	41	4.50	3.47	−18
p60c (p)	46	5.50	2.54	−38
rub (u)	53	5.90	6.12	−8
Target protein and its fusion forms ^1^				
CAT3	511	59.00	6.71	−4
rCAT3	550	63.22	5.80	−18
tCAT3	554	63.75	5.62	−22
pCAT3	559	64.75	4.92	−42
uCAT3	566	65.14	6.12	−12

^1^ All contain additional myc-tag (EQKLISEEDL) at the N-terminal and 8 amino acids (L, E plus 6x His) at the C-terminal.

## Data Availability

All data supporting the conclusions of this article are included within the article and the Supplementary Materials.

## Supplementary Materials

The following supporting information can be downloaded at: https://www.mdpi.com/article/10.3390/ijms252212181/s1. Reference [71] is cited in the supplementary materials.
