# Supplementary material for: Potentiation of Catalase-Mediated Plant Thermotolerance by N-Terminal Attachment of Solubilizing/Thermostabilizing Fusion Partners
Source: Int J Mol Sci. 2024 Nov 13;25(22):12181. doi: 10.3390/ijms252212181 (PMC11594932; doi:10.3390/ijms252212181)
Supplement: Supplementary file 1 [file ijms-25-12181-s001.zip › ijms-3290156-supplementary.pdf]

Supplementary Materials

Table S1. Primers used in this study

Name	Sequence (5′ →3′)
AtCAT3-5Nd	TAAGAAGGAGATATACATATGGGTGAACAAAAGCTCATCTCAGAAGAGGATCTTGATCC TTACAAGTATCGTCC
AtCAT3-3Xh	GTGGTGGTGGTGGTGCTCGAGGATGCTTGGTCTCACGTTCAG
PetNd-uF	GTTTAACTTTAAGAAGGAGATATAC
tCAT3-R	GAGCTTTTGTTCACCCATATGGTACTCCTCTCCTTC
rCAT3-R	GAGCTTTTGTTCACCCATATGTTCATACTCTTCAGATC
uCAT3-R	GAGCTTTTGTTCACCCATATGATCTTCTAACTTTTCAAATTCAC
RubNd-F	TAAGAAGGAGATATACATATGGCTAAGTGGGTTTGTAAGATATG
P60c-Fin	CCTTGATGATGAAGATGATGATGAAGAGGATGAGGATGACGAAGACGGTGAAGAAG
P60c-Rin	TCATCATCATCCATTTCGTCTTCATCTTCTTCATCATCTTCTTCACCGTCTTCGTC
P60c-Fou	GCGTACTCGAGGACATGGATGATGAGGATGATGACCTTGATGATGAAGATGATG
P60c-Rou	GGCAGTCTCGAGTTCATCGTAATTATATCCATCATCATCATCATCCATTTCGTC
PbNd-F	TAAGAAGGAGATATACATATGGACATGGATGATGAGGATG
pCAT3-R	GAGCTTTTGTTCACCCATATGTTCATCGTAATTATATCCATC
Pt7Up-Fw	ACCGCGAAATTAATACGACTCAC
CAT3y-R	TACATGATGCGGCCCTCTAGATCAGATGCTTGGCCTCACGTTC
CAT3y-F	GGGAATATTAAGCTTGGTACCGACGCAACCATGGGTGAACAAAAGCTC
rCAT3y-F	GGGAATATTAAGCTTGGTACCGACGCAACCATGGACCAAGGTGCTCA
tCAT3y-F	GGGAATATTAAGCTTGGTACCGACGCAACCATGGGTGAGGGTATGGA
pCAT3y-F	GGGAATATTAAGCTTGGTACCGACGCAACCATGGACATGGATGATGAG
uCAT3y-F	GGGAATATTAAGCTTGGTACCGACGCAACCATGGCTAAGTGGGTTTG
pYES2-Rv	GGGACCTAGACTTCAGGTTGTC
CAT3-5F	GACACGCTGGAATTCTAGTATACTAAACCATGGGTGAACAAAAGCTCATC
tCAT3-5F	GACACGCTGGAATTCTAGTATACTAAACCATGGGTGAGGGTATGGAAG
rCAT3-5F	GACACGCTGGAATTCTAGTATACTAAACCATGGACCAAGGTGCTCAAC
pCAT3-5F	GACACGCTGGAATTCTAGTATACTAAACCATGGACATGGATGATGAGGA
uCAT3-5F	GACACGCTGGAATTCTAGTATACTAAACCATGGCTAAGTGGGTTTGTAAG
CAT3-3Xp	AGCTCAGTAGCAATTCTCGAGTCAGATGCTTGGTCTCACGTTCAG
VecEn-5Xb	GAGAACACGGGGGACTCTAGACACGCTGGAATTCTAGTATAC
PetHis-dRv	GCTTTGTTAGCAGCCGGATCTCAG
35sP-Sq	GACGCACAATCCCACTATCCTTC
NosDw-nR	GTGCTGCAAGGCGATTAAGTTG
Nt18S-iFw	GAAACGGCTACCACATCCAAG
Nt18S-iRv	GGCAAATGCTTTCGCAGTTG
Myc-Fw	GGTGAACAAAAGCTCATCTCAG
CAT3-iRv	TCCCTCATGGTTTCAGGACTG

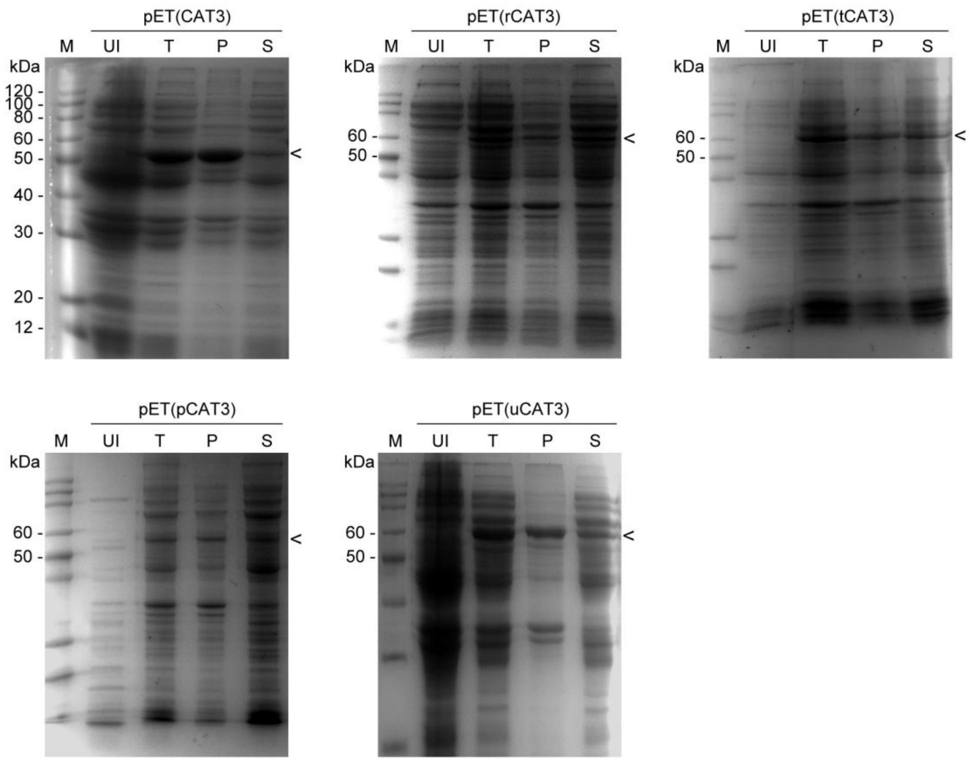

Figure S1. Recombinant expression in *E. coli* of CAT3 and its various fusion proteins (r/t/p/uCAT3) by SDS-PAGE analysis. M: protein size marker. UI, T: the total lysates of pre-induced and induced bacterial cells, respectively. S, P: the supernatant and pellet of ‘T’ fractionated by centrifugation, respectively. ‘<’ indicate the expressed target proteins.

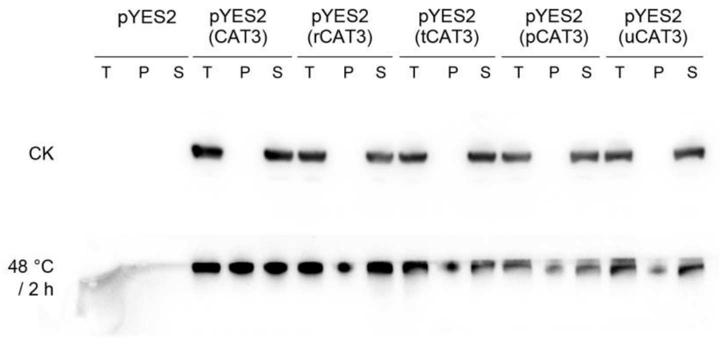

Figure S2. Immunoblotting detection of the solubility changes of CAT3 and its fusion forms (r/t/p/uCAT3) expressed in recombinant yeast (*S. cerevisiae* INVSC1) strains after heat stress (48 °C/ 2 h). CK: before heat treatment; T: total proteins in an aliquot of the cell lysate of yeast cells. S, P: the supernatant and pellet fraction of ‘T’ by centrifugation, respectively.

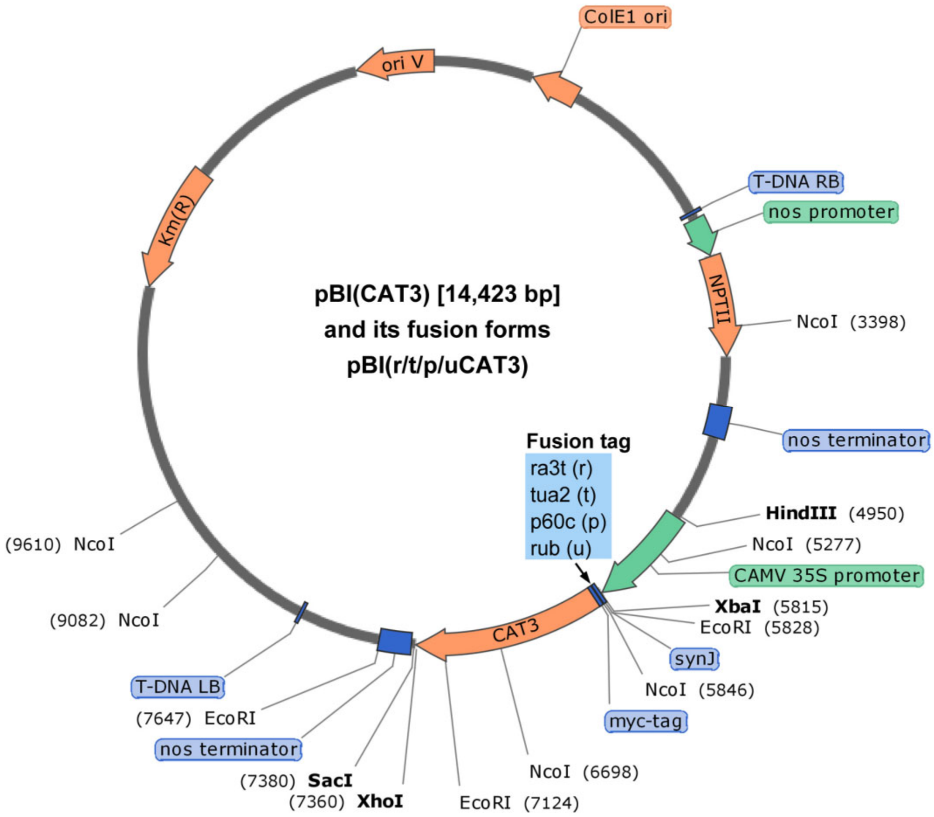

Figure S3. The structural diagram (drawn by SnapGene program) of plant expression vectors pBI(CAT3) and its various fusion forms pBI(r/t/p/uCAT3) derived from pBI121. Fusion tags (r, t, p, u) are located ahead of myc-tag. LB: left border; RB: right border. synJ: an artificial translation enhancer [Reference 71].

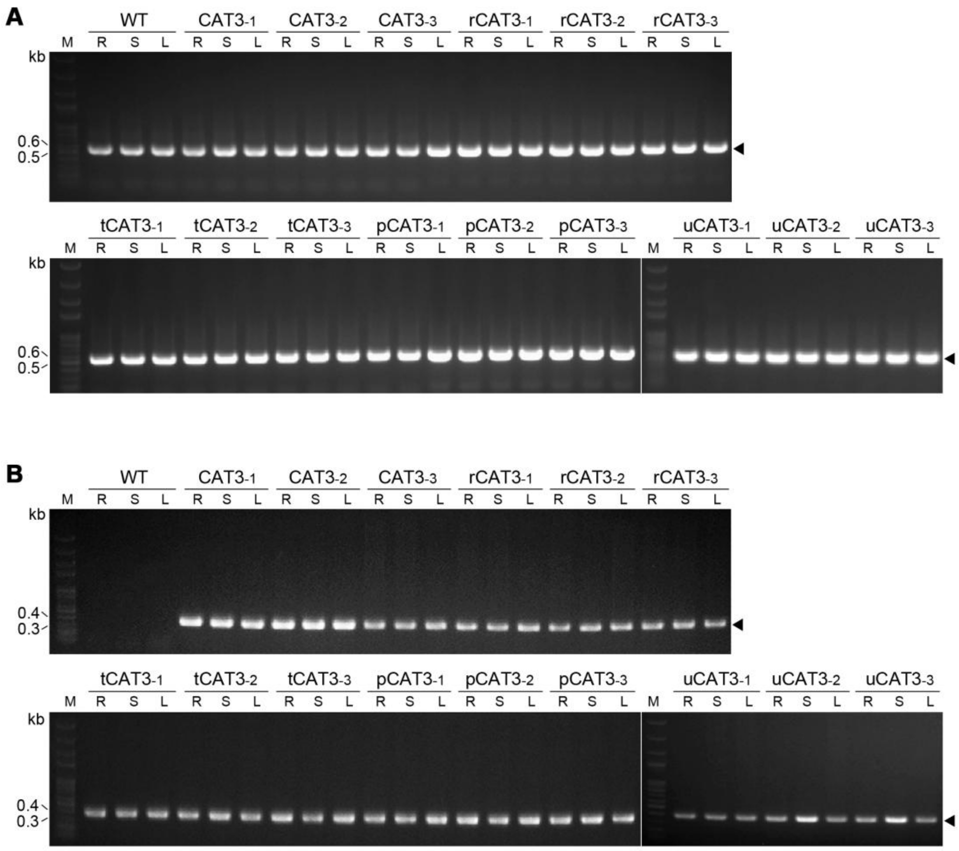

Figure S4. Target gene expression in the main tissues of transgenic tobacco plants analyzed by RT-PCR. **(A)** the internal reference gene *18S rRNA*, with a 552 bp product amplified by specific primer-pair Nt18S-iFw/Nt18S-iRv. **(B)** various *CAT3* transgenes, with a 383 bp product amplified by the common primer-pair Myc-Fw/CAT3-iRv. R, S, L: root, stem, and leaf, respectively. Arrow-heads indicate the target products.

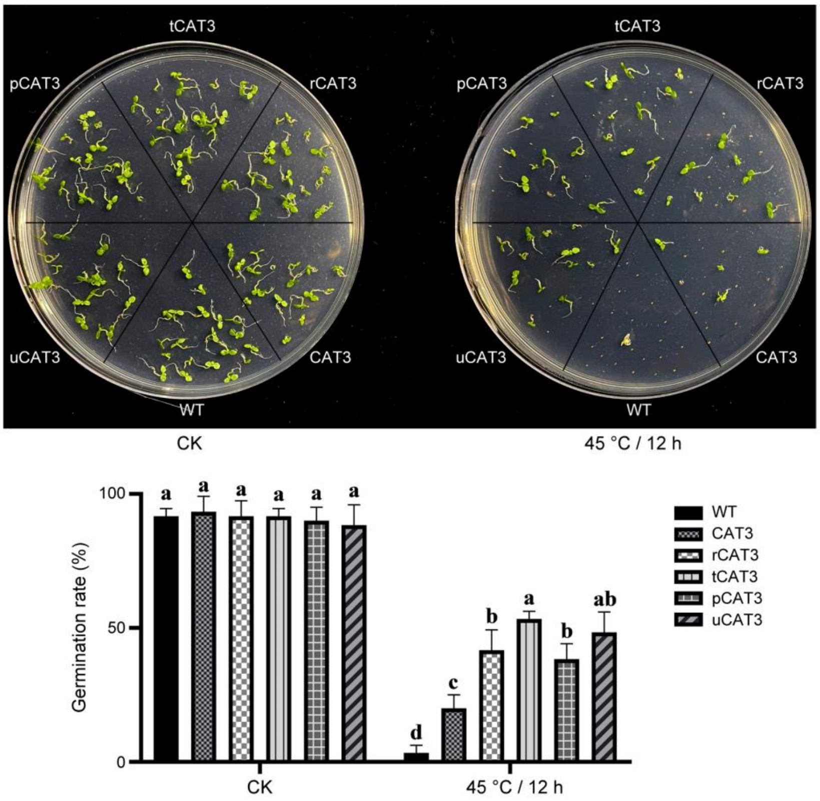

Figure S5. Germination comparison of WT and various CAT3 transgenic tobacco seeds after a heat stress of 12 h at 45 °C. CK: before heat treatment.

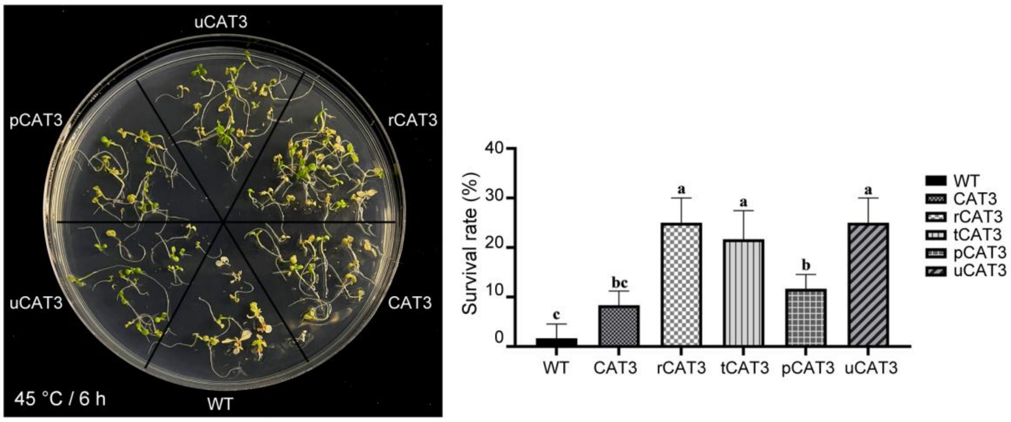

Figure S6. Survival comparison of WT and various CAT3 transgenic tobacco seedlings after a heat stress of 6 h at 45 °C.
